# Computed tomography-measured body composition can predict long-term outcomes for stage I-III colorectal cancer patients

**DOI:** 10.3389/fonc.2024.1420917

**Published:** 2024-07-08

**Authors:** Han Zhou, Lei Tian, Yiting Wu, Sibin Liu

**Affiliations:** Radiology Department, Jingzhou Hospital Affiliated to Yangtze University, Jingzhou, Hubei, China

**Keywords:** colorectal cancer, CT, biomarkers, body composition, prognosis

## Abstract

**Background:**

There remains a pressing need to identify biomarkers capable of reliably predicting prognostic outcomes for colorectal cancer (CRC) patients. As several body composition parameters have recently been reported to exhibit varying levels of prognostic significance in particular cancers, the present study was devised to assess the ability of body composition to predict long-term outcomes for CRC patients with different stages of disease.

**Methods:**

In total, this retrospective analysis enrolled 327 stage I-III CRC patients whose medical records were accessed for baseline demographic and clinical data. Primary outcomes for these patients included disease-free and overall survival (DFS and OS). The prognostic performance of different musculature, visceral, and subcutaneous fat measurements from preoperative computed tomography (CT) scans was assessed.

**Results:**

Over the course of follow-up, 93 of the enrolled patients experienced recurrent disease and 39 died. Through multivariate Cox regression analyses, the visceral/subcutaneous fat area (V/S) ratio was found to be independently associated with patient DFS (HR=1.93, 95% CI: 1.24–3.01, P=0.004), and the skeletal muscle index (SMI) as an independent predictor for OS (HR=0.43, 95% CI: 0.21–0.89, P=0.023). Through subgroup analyses, higher V/S ratios were found to be correlated with reduced DFS among patients with stage T3/4 (P=0.011), lymph node metastasis-positive (P=0.002), and TNM stage III (P=0.002) disease, whereas a higher SMI was associated with better OS in all T stages (P=0.034, P=0.015), lymph node metastasis-positive cases (P=0.020), and in patients with TNM stage III disease (P=0.020).

**Conclusion:**

Both the V/S ratio and SMI offer potential utility as clinical biomarkers associated with long-term CRC patient prognosis. A higher V/S ratio and a lower SMI are closely related to poorer outcomes in patients with more advanced disease.

## 1 Introduction

Colorectal cancer (CRC) is characterized by high, steadily rising rates of global incidence and mortality (1, 2), yet biomarkers capable of predicting the long-term outcomes of affected patients are still lacking. Quantitative measurements of skeletal muscle mass and adipose tissue distributions have recently been demonstrated to offer significant prognostic utility in CRC and many other forms of cancer, leading to a growing body of research focused on how body composition relates to oncological outcomes.

Chronic inflammation is a major driver of carcinogenesis (3). Sarcopenia is a condition defined by declines in the function and mass of skeletal muscle. Cancer-associated muscle loss primarily results from systemic inflammatory responses induced by host-tumor interactions (4), as inflammatory cytokines derived from tumor cells such as IL-6 and TNF-α can impair the differentiation of skeletal muscle cells (5), interfere with insulin signaling, and thereby drive more severe insulin resistance and the degradation of muscle tissue (6). Insulin resistance can be further exacerbated by sarcopenia as well (7, 8), and the low-grade tumor-induced inflammatory activity can provoke inflammatory activity within the muscles, contributing to systemic inflammatory activity and additional muscle degradation (9). Adipose tissue serves as an endocrine organ that is vital for the control of metabolic and energy homeostasis such that when it accumulates at excessively high levels, this can trigger the release of inflammatory cytokines and adipokines related to CRC progression (10). Given its proximity to nearby organs, visceral adipose tissue can transfer lipids to these nearby tissues, leading to altered metabolic activity that can be conducive to oncogenic growth (10, 11).

Abdominopelvic computed tomography (CT) is an imaging strategy that is routinely employed for the management and initial staging of patients with CRC, and it can provide accurate information regarding muscle mass and the presence of adipose tissue (12). Quantitative measures of adipose tissue distribution and skeletal muscle mass have both been linked to poor prognostic outcomes in patients with CRC (7, 8, 13–24). Efforts to evaluate and improve body composition either before tumor development or after disease onset may provide opportunities for novel therapeutic interventions.

Both exercise (25) and nutritional support (26, 27) have previously been demonstrated to have positive effects on body composition and other physiological changes that may protect against the development of CRC. While there have been some prior efforts to examine the relevance of body composition to CRC patient outcomes, the results of these studies have been inconsistent and relatively little is known with respect to how these parameters vary as a function of tumor staging. The present study was conducted based on the hypothesis that body composition measurements can provide valuable insights into prognostic outcomes across different stages of CRC, particularly in patients with more advanced disease. The specific goal of these analyses was to probe the relationship between a range of body composition parameters and both overall and disease-free survival (OS and DFS) in CRC patients with stage I-III disease, validating these analyses in different tumor stages.

## 2 Materials and methods

### 2.1 Study population

**Figure 1:**
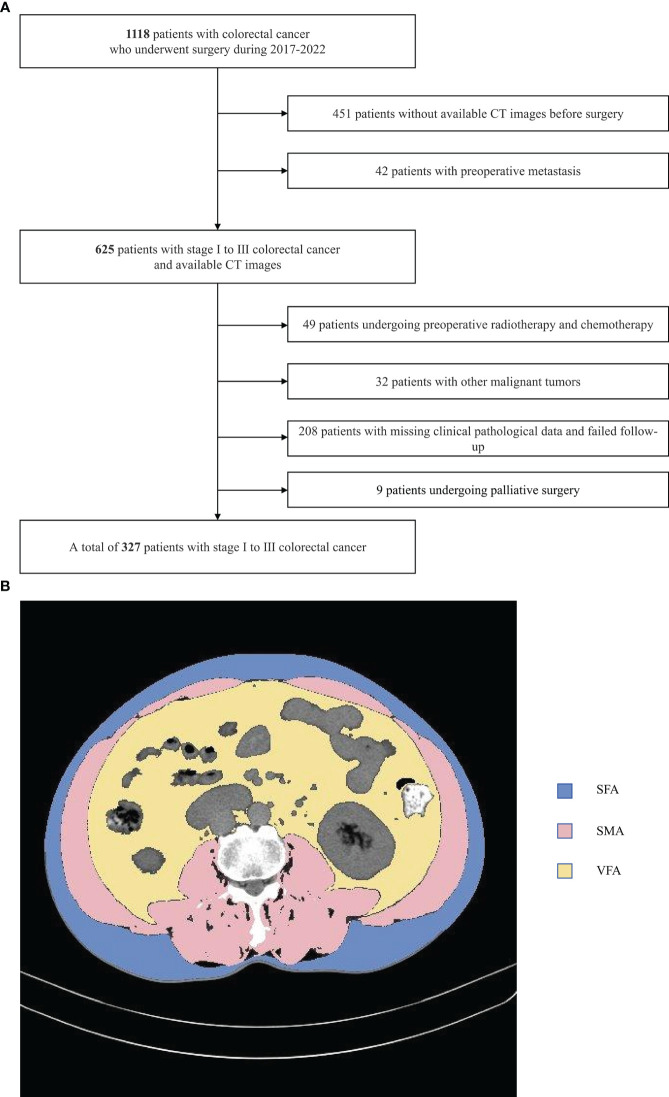
Study flow chart **(A)** and CT-based measures of body composition **(B)** subcutaneous fat area (SFA),visceral fat area (VFA) and skeletal muscle area(SMA).

This was a retrospective analysis of patients with stage I-III CRC who underwent surgical treatment in the Colorectal Surgery Department of Jingzhou Hospital affiliated with Yangtze University between January 2017 and December 2021 (**Figure 1A**). To be eligible for inclusion, patients needed to (1) have a pathological diagnosis of primary CRC; (2) have undergone an abdominal CT scan within a 30-day period prior to surgery; and (3) have a complete set of clinical and follow-up data. Patients were excluded if they (1) had been diagnosed with other malignancies; (2) had preoperatively undergone chemotherapy or radiotherapy; (3) exhibited any preoperative metastases; (4) were missing any clinicopathological or follow-up data; (5) had undergone palliative surgery. Tumor staging was assessed by pathologists as per the 8th edition AJCC TNM staging guidelines.

The studies involving human participants were reviewed and approved by [Ethics Committee of Jingzhou Hospital affiliated with Yangtze University]. As this study was retrospective in nature, the need for informed consent was waived.

### 2.2 Clinical data

Medical records were accessed to obtain patient clinical and demographic characteristics including age, gender, BMI, American Society of Anesthesiologists (ASA) classification, diabetes, hypertension, surgical approach, and postoperative chemotherapy. In addition, preoperative blood test results for serum CEA and CA199 levels were also analyzed, as were clinicopathological features such as tumor location, tumor differentiation, pathological tumor type, and tumor T stage, N stage, and TNM staging.

### 2.3 Clinical outcomes

Primary endpoints for this study were OS and DFS. OS was measured from tumor resection to death or most recent follow-up, while DFS was measured from tumor resection to metastasis, recurrence, death, or most recent follow-up. Follow-up was primarily conducted through surveys administered in an outpatient setting or phone-based contact with patients.

### 2.4 Body composition measurements

ImageJ v1.52 (NIH, USA) was used to analyze all images, conducting semi-automated tissue segmentation with the following HU thresholds: adipose tissue (-190 to -30), skeletal muscle (-29 to 150) (**Figure 1B**). This approach allowed for the measurement of skeletal muscle, visceral fat, and subcutaneous fat area values (in cm²), together with the calculation of the visceral to subcutaneous fat area (V/S) ratio. The measured area values were normalized to patient height (m²), enabling the calculation of the subcutaneous fat index (SFI), visceral fat index (VFI), and skeletal muscle index (SMI) values (in cm²/m²). The average radiodensity values for subcutaneous fat (SFD), visceral fat (VFD), and skeletal muscle (SMD) were measured using equally sized regions of interest.

### 2.5 Statistical analysis

Given the lack of standardized cutoff values for various body composition metrics, particularly in Asian populations, we derived the cutoff values used in this study from the median values of our sample data to divide patients into high and low groups. A radiologist blinded to patient characteristics conducted all measurements.

Continuous data are reported as means ± SD or medians and interquartile ranges (IQRs), while categorical variables are presented as numbers and percentages. Continuous data were compared with Mann-Whitney U-tests or Student’s t-tests, while categorical variables were compared using Fisher’s exact test or chi-square tests. Nonlinear associations between body composition and patient survival were assessed with restricted cubic spline models. Variables related to the duration of patient survival were identified through univariate and multivariate analyses, with those variables that exhibited a P < 0.1 being incorporated into the multivariate analysis, calculating hazard ratios (HRs) with 95% confidence intervals (CIs). ROC curves were used for model evaluation, while survival analyses for the overall patient cohort and particular subgroups were performed with Kaplan-Meier curves and log-rank tests. Data were analyzed in R 4.3.0 (2023–04-21) and Zstats (www.medsta.cn/software). P<0.05 served as the cutoff for significance.

## 3 Results

### 3.1 Baseline characteristics

In total, 1118 patients were evaluated for study inclusion, of whom 327 met with the criteria for enrollment in these analyses. These 327 patients exhibited an average age of 60.9 ± 10.6 years, 62.7% were male, and the median duration of follow-up was 48.7 (40.2–60.2) months. Over the course of follow-up, 93 of these 327 patients developed recurrent disease at a median recurrence time of 45.1 (36.9–59.1) months, while 39 died with a median of 30.0 (21.0–40.0) months to death. CA199 and CEA levels in the recurrence group were significantly higher than those for patients who did not experience recurrent disease (P<0.001, P=0.001). The distributions of diabetes (P=0.035), T stage (P<0.001), N stage (P<0.001), and TNM stage (P<0.001) differed significantly between patients who did and did not develop recurrent CRC. Of the analyzed body composition parameters, only the V/S ratio differed significantly between these two groups of patients (P=0.020). For further details, see **Table 1**.

**Table 1:** Demographic and clinical variables.

Variables	Total (n = 327)	Recurrence		P
		No (n = 234)	Yes (n = 93)	
Age	60.9 ± 10.6	60.3 ± 10.7	62.7 ± 10.3	0.061
BMI >25 (kg/m²)				0.213
No	256 (78.3)	179 (76.5)	77 (82.8)	
Yes	71 (21.7)	55 (23.5)	16 (17.2)	
CEA (ng/ml)	3.6 (2.1, 7.5)	3.1 (1.7, 6.0)	5.2 (2.7, 10.8)	<.001
CA199(U/ml)	10.6 (6.1, 21.6)	9.9 (5.6, 18.3)	14.8 (8.1, 27.4)	0.001
Gender				0.090
Women	122 (37.3)	94 (40.2)	28 (30.1)	
Men	205 (62.7)	140 (59.8)	65 (69.9)	
ASA score				0.667
I–II	122 (37.3)	89 (38.0)	33 (35.5)	
III–IV	205 (62.7)	145 (62.0)	60 (64.5)	
TNM stage				<.001
1	60 (18.4)	56 (23.9)	4 (4.3)	
2	149 (45.6)	117 (50.0)	32 (34.4)	
3	118 (36.1)	61 (26.1)	57 (61.3)	
T stage				<.001
1/2	73 (22.3)	65 (27.8)	8 (8.6)	
3/4	254 (77.7)	169 (72.2)	85 (91.4)	
N stage				<.001
0	209 (63.9)	173 (73.9)	36 (38.7)	
1	80 (24.5)	43 (18.4)	37 (39.8)	
2	38 (11.6)	18 (7.7)	20 (21.5)	
Histotype				0.673
Nonadenocarcinoma	28 (8.6)	21 (9.0)	7 (7.5)	
Adenocarcinoma	299 (91.4)	213 (91.0)	86 (92.5)	
Differentiation				0.867
Well–moderate	276 (84.4)	198 (84.6)	78 (83.9)	
Poor	51 (15.6)	36 (15.4)	15 (16.1)	
Operating mode				0.372
Open	28 (8.6)	18 (7.7)	10 (10.8)	
Laparoscopic	299 (91.4)	216 (92.3)	83 (89.3)	
Adjuvantchemo therapy				0.494
No	86 (26.3)	64 (27.4)	22 (23.7)	
Yes	241 (73.7)	170 (72.7)	71 (76.3)	
Hypertension				0.842
No	233 (71.3)	166 (70.9)	67 (72.0)	
Yes	94 (28.8)	68 (29.1)	26 (28.0)	
Diabetes				0.035
No	287 (87.8)	211 (90.2)	76 (81.7)	
Yes	40 (12.2)	23 (9.8)	17 (18.3)	
Tumor location				0.319
Rectum	176 (53.8)	130 (55.6)	46 (49.5)	
Colon	151 (46.2)	104 (44.4)	47 (50.5)	
SFI, cm²/m²				0.820
Low	162 (49.5)	115 (49.2)	47 (50.5)	
High	165 (50.5)	119 (50.9)	46 (49.5)	
VFI, cm²/m²				0.495
Low	168 (51.4)	123 (52.6)	45 (48.4)	
High	159 (48.6)	111 (47.4)	48 (51.6)	
SMI, cm²/m²				0.541
Low	160 (48.9)	112 (47.9)	48 (51.6)	
High	167 (51.1)	122 (52.1)	45 (48.4)	
SFD, HU				0.473
Low	165 (50.5)	121 (51.7)	44 (47.3)	
High	162 (49.5)	113 (48.3)	49 (52.7)	
VFD, HU				0.637
Low	162 (49.5)	114 (48.7)	48 (51.6)	
High	165 (50.5)	120 (51.3)	45 (48.4)	
SMD, HU				0.391
Low	160 (48.9)	111 (47.4)	49 (52.7)	
High	167 (51.1)	123 (52.6)	44 (47.3)	
V/S ratio				0.020
Low	167 (51.1)	129 (55.1)	38 (40.9)	
High	160 (48.9)	105 (44.9)	55 (59.1)	

BMI, body mass index; CEA, carcinoembryonic antigen; CA199, cancer antigen 199; ASA, American Society of Anesthesiologists; BMI, body mass index; TNM, tumor-node-metastasis; SFI, subcutaneous fat index; VFI, visceral fat index; SMI, skeletal muscle index; SFD, subcutaneous fat radiodensity; VFD, visceral fat radiodensity; SMD, skeletal muscle radiodensity; HU, Hounsfield Unit; V/S ratio, visceral fat area to subcutaneous fat area ratio.

### 3.2 Correlation between BMI and body composition

Among the fat-related indicators, BMI was found to be correlated with VFA and SFA. Specifically, BMI and VFA showed a positive correlation (rs=0.666, P < 0.001) (**Supplementary Figure 1A**), while BMI and SFA exhibited a moderate positive correlation (rs=0.539,P < 0.001) (**Supplementary Figure 1B**). In contrast, no correlation was found between BMI and the V/S ratio (rs=0.237, P < 0.001) (**Supplementary Figure 1C**). Furthermore, SMI and the V/S ratio showed a weak positive correlation (rs=0.331, P < 0.001) (**Supplementary Figure 1D**).

### 3.3 Testing for non-linear associations between body composition parameters and long-term outcomes

**Figure 2:**
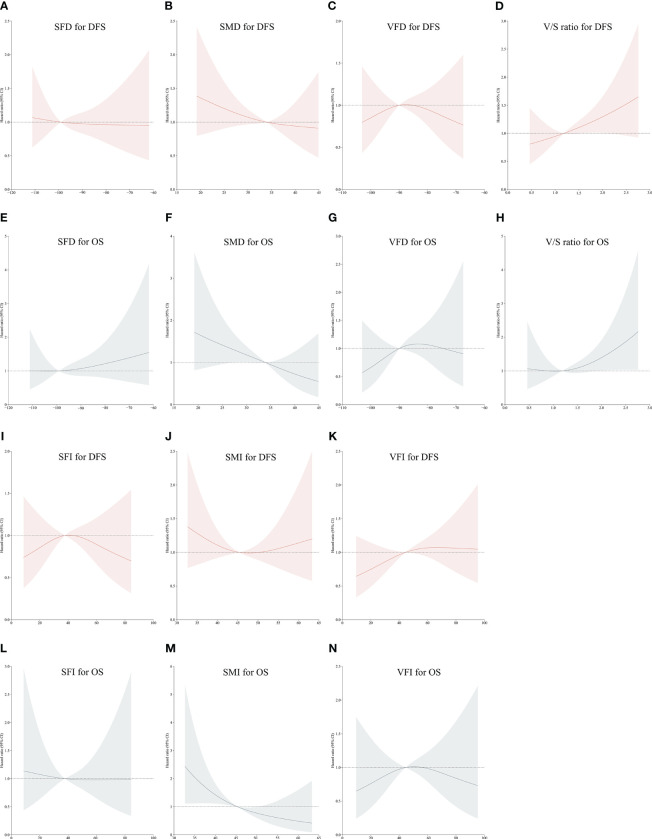
Restricted Cubic Spline curves corresponding to disease-free or overall survival (DFS or OS) based on patient SFD **(A, E)**, SMD **(B, F)**, VFD **(C, G)**, V/S ratio values **(D, H)**, SFI **(I, L)**, SMI **(J, M)** and VFI **(K, N)**. Red portions of the curves correspond to DFS, while gray portions correspond to OS.

Using restricted cubic spline (RCS) curve analyses, body composition parameters were found to be linearly associated with the OS and DFS of CRC patients. In particular, an increase in SMD was associated with corresponding reductions in the DFS hazard ratio (**Figure 2B**), whereas as the V/S ratio rose, a linear increase in the hazard ratio for DFS was observed (**Figure 2D**).

A linear decrease in the hazard ratio for OS was observed with increasing SMI (**Figure 2M**) and SMD (**Figure 2F**). A linear increase in the hazard ratio for OS was also observed with increasing V/S ratio values (**Figure 2H**). However, no significant trends were found in other body compositions concerning the long-term prognosis of colorectal cancer (**Figures 2A, C, E, G, I–L, N**).

### 3.4 The relationship between body composition parameters and prognostic outcomes in the overall patient cohort

**Figure 3:**
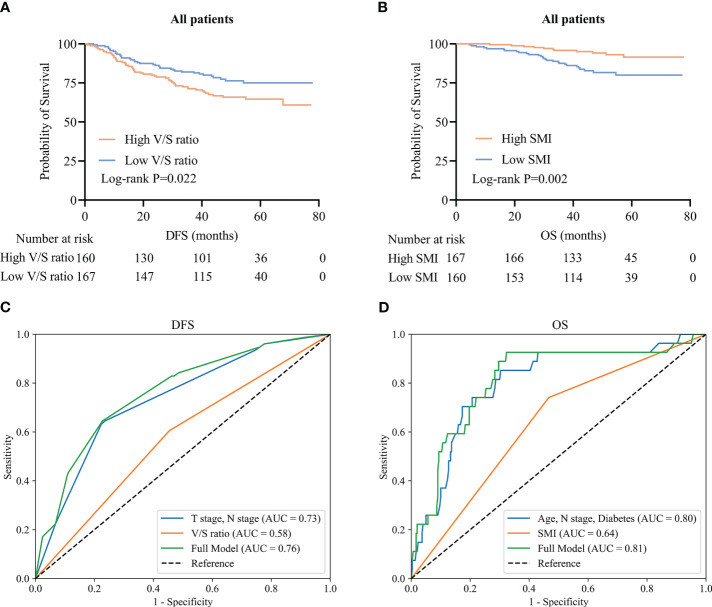
CRC patient survival and time-dependent ROC curves. **(A, B)** Kaplan-Meier DFS **(A)** and OS **(B)** curves for the overall CRC patient population. **(C, D)** Time-dependent ROC curves for patient DFS **(C)** and OS **(D)**.

To clarify which body composition parameters were significantly related to certain clinical outcomes, univariate and multivariate Cox regression analyses of OS and DFS were conducted (**Table 2**). Of the 327 patients with stage I-III disease in this study, recurrence affected 93 (28.4%), with a median time to recurrence of 29.9 (21.0–39.5) months. In multivariate analyses, the V/S ratio was identified as the only measure of body composition that was independently associated with patient DFS. Specifically, the risk of recurrent disease was higher among patients with higher V/S ratios as compared to patients with lower V/S ratios (HR=1.93, 95% CI: 1.24–3.01, P=0.004). Kaplan-Meier curves confirmed that the DFS of patients with a high V/S ratio was significantly reduced as compared to patients exhibiting lower V/S ratio values (44.9 (30.4–58.0) vs 45.4 (38.8–59.4), P=0.022) (**Figure 3A**). ROC curves additionally suggested the potential for the utility of the V/S ratio as a supplementary component of the overall model, providing for superior predictive accuracy (**Figure 3C**).

**Table 2:** Univariable and multivariable Cox regression analyses for disease-free survival and overall survival.

Variables	Disease-free survival				Overall survival			
	Univariable analysis		Multivariable analysis		Univariable analysis		Multivariable analysis	
	HR (CI 95%)	P value	HR (CI 95%)	P value	HR (CI 95%)	P value	HR (CI 95%)	P value
Age	1.02 (1.00 ~ 1.04)	0.085	1.02 (1.00 ~ 1.04)	0.139	1.05 (1.02 ~ 1.08)	0.005	1.05 (1.01 ~ 1.08))	0.010
Gender								
Women	1.00 (Reference)				1.00 (Reference)			
Men	1.42 (0.91 ~ 2.22)	0.119			1.18 (0.61 ~ 2.29)	0.629		
BMI >25(kg/m²)								
No	1.00 (Reference)				1.00 (Reference)			
Yes	0.75 (0.44 ~ 1.29)	0.296			0.52 (0.20 ~ 1.33)	0.171		
ASA score								
I–II	1.00 (Reference)				1.00 (Reference)			
III–IV	1.11 (0.73 ~ 1.70)	0.621			1.12 (0.58 ~ 2.15)	0.739		
CEA (ng/ml)	1.01 (1.01 ~ 1.01)	<.001	1.00 (1.00 ~ 1.01)	0.139	1.01 (1.01 ~ 1.02)	0.003	1.00 (1.00~ 1.01)	0.596
CA199 (U/ml)	1.00 (1.00 ~ 1.00)	0.185			1.00 (1.00 ~ 1.00)	0.573		
T stage								
1/2	1.00 (Reference)		1.00 (Reference)		1.00 (Reference)		1.00 (Reference)	
3/4	3.58 (1.73 ~ 7.40)	<.001	2.80 (1.34 ~ 5.85)	0.006	3.74 (1.15 ~ 12.14)	0.028	2.30 (0.70 ~ 7.60)	0.172
N stage								
0	1.00 (Reference)		1.00 (Reference)		1.00 (Reference)		1.00 (Reference)	
1	3.41 (2.15 ~ 5.41)	<.001	3.50 (2.17 ~ 5.65)	<.001	2.91 (1.39 ~ 6.11)	0.005	2.64 (1.23 ~ 5.67)	0.013
2	4.71 (2.72 ~ 8.17)	<.001	4.79 (2.70 ~ 8.52)	<.001	5.85 (2.64 ~ 12.97)	<.001	8.72 (3.61 ~ 21.05)	<.001
Histotype								
Nonadenocarcinoma	1.00 (Reference)				1.00 (Reference)			
Adenocarcinoma	1.27 (0.59 ~ 2.75)	0.539			1.15 (0.35 ~ 3.73)	0.819		
Differentiation								
Well–moderate	1.00 (Reference)				1.00 (Reference)			
Poor	1.04 (0.60 ~ 1.81)	0.892			1.22 (0.54 ~ 2.77)	0.629		
Operating mode								
Open	1.00 (Reference)				1.00 (Reference)			
Laparoscopic	0.73 (0.38 ~ 1.40)	0.341			0.51 (0.21 ~ 1.22)	0.132		
Adjuvant chemotherapy								
No	1.00 (Reference)				1.00 (Reference)			
Yes	1.23 (0.76 ~ 1.98)	0.405			0.70 (0.36 ~ 1.36)	0.292		
Hypertension								
No	1.00 (Reference)				1.00 (Reference)			
Yes	0.94 (0.60 ~ 1.48)	0.797			1.57 (0.82 ~ 2.99)	0.172		
Diabetes								
No	1.00 (Reference)		1.00 (Reference)		1.00 (Reference)		1.00 (Reference)	
Yes	1.76 (1.04 ~ 2.97)	0.036	1.67 (0.95 ~ 2.93)	0.077	3.11 (1.55 ~ 6.25)	0.001	3.13 (1.47 ~ 6.66)	0.003
Tumor location								
Rectum	1.00 (Reference)				1.00 (Reference)			
Colon	1.17 (0.78 ~ 1.75)	0.455			1.36 (0.72 ~ 2.55)	0.341		
SFI (cm^2^/m^2^)								
Low	1.00 (Reference)				1.00 (Reference)			
High	0.93 (0.62 ~ 1.39)	0.707			0.91 (0.49 ~ 1.70)	0.766		
VFI (cm^2^/m^2^)								
Low	1.00 (Reference)				1.00 (Reference)			
High	1.10 (0.73 ~ 1.65)	0.643			1.08 (0.58 ~ 2.03)	0.799		
SMI (cm^2^/m^2^)								
Low	1.00 (Reference)				1.00 (Reference)		1.00 (Reference)	
High	0.86 (0.57 ~ 1.29)	0.457			0.34 (0.17 ~ 0.69)	0.003	0.43 (0.21 ~ 0.89)	0.023
SFD (HU)								
Low	1.00 (Reference)				1.00 (Reference)			
High	1.13 (0.75 ~ 1.69)	0.569			1.14 (0.60 ~ 2.13)	0.691		
VFD (HU)								
Low	1.00 (Reference)				1.00 (Reference)			
High	0.90 (0.60 ~ 1.35)	0.600			1.03 (0.55 ~ 1.92)	0.937		
SMD (HU)								
Low	1.00 (Reference)				1.00 (Reference)			
High	0.85 (0.56 ~ 1.27)	0.427			0.59 (0.31 ~ 1.13)	0.110		
V/S ratio								
Low	1.00 (Reference)		1.00 (Reference)		1.00 (Reference)			
High	1.62 (1.07 ~ 2.44)	0.023	1.93 (1.24 ~ 3.01)	0.004	1.56 (0.83 ~ 2.96)	0.169		

For risk factors with more than two categories, the first category was considered as the reference group.

HR, hazard ratio; CI, confidence interval; BMI, body mass index; CEA, carcinoembryonic antigen; CA199, cancer antigen 199; ASA, American Society of Anesthesiologists; BMI, body mass index; TNM, tumor-node-metastasis; SFI, subcutaneous fat index; VFI, visceral fat index; SMI, skeletal muscle index; SFD, subcutaneous fat radiodensity; VFD, visceral fat radiodensity; SMD, skeletal muscle radiodensity; HU, Hounsfield Unit; V/S ratio, visceral fat area to subcutaneous fat area ratio.

Of the included patients, 39 had died as of the follow-up cut-off with a median of 30.0 (21.0–40.0) months to death. Following adjustment for clinical factors, SMI was identified as an independent predictor of OS (HR=0.43, 95% CI: 0.21–0.89, P=0.023). Kaplan-Meier curves indicated that patients with low SMI values tended to exhibit shorter OS than patients with high SMI values (30.0 (20.0–37.0) vs 33.0 (25.0–44.0); P=0.002) (**Figure 3B**). ROC curves indicated that the incorporation of SMI into comprehensive clinical analyses provided improved model accuracy when seeking to predict OS (**Figure 3D**).

### 3.5 The relationship between body composition parameters and prognostic outcomes across T staging subgroups

**Figure 4:**
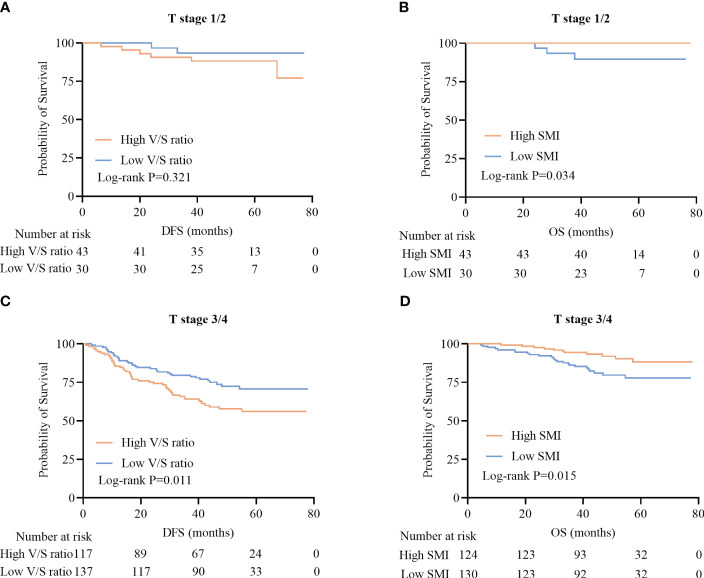
Survival curves for CRC patients stratified according to T stage. **(A, B)** Kaplan-Meier DFS **(A)** and OS **(B)** curves for patients with stage T1/2 disease. **(C, D)** Kaplan-Meier DFS **(C)** and OS **(D)** curves for patients with stage T3/4 disease.

Among patients with T1/2 disease, DFS did not differ significantly as a function of V/S ratio (P=0.321) (**Figure 4A**), whereas among patients with more advanced T staging (T3/4), a high V/S ratio was associated with significantly worse DFS as compared to that of individuals with a low V/S ratio (41.5 (24.4–57.3) vs 44.4 (38.2–59.4), P=0.011) (**Figure 4C**). Irrespective of T staging (T1/2 or T3/4), patients with a high SMI presented with significantly better OS as compared to that of patients with a low SMI (P=0.034; 33.0 (25.0–44.0) vs 30.0 (17.0–37.0), P=0.015) (**Figures 4B, D**).

### 3.6 The relationship between body composition parameters and prognostic outcomes across N staging subgroups

**Figure 5:**
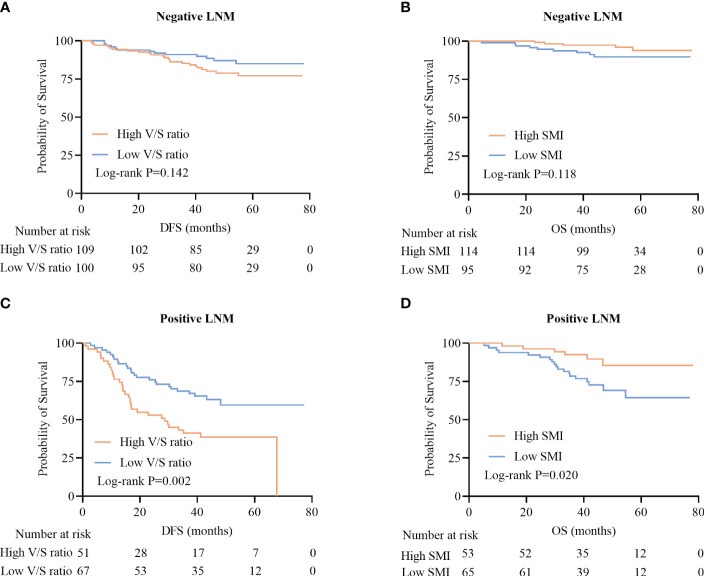
Survival curves for CRC patients stratified according to lymph node metastasis (LNM). **(A, B)** Kaplan-Meier DFS curves for LNM-negative patients **(A)**, OS curves for LNM-negative patients **(B)**, **(C, D)** DFS curves for LNM-positive patients **(C)**, and OS curves for LNM-positive patients **(D)**.

Among patients positive for lymph node metastasis (LNM), high V/S ratio values were associated with significantly worse DFS as compared to that of patients with low V/S ratio values (28.7 (13.3–41.7) vs 41.3 (25.6–49.9), P=0.002) (**Figure 5C**). LNM-positive patients with lower SMI values presented with significantly worse OS as compared to patients with higher SMI values (31.0 (23.0–36.0) vs 32.0 (22.0–40.0), P=0.020) (**Figure 5D**). In contrast, DFS and OS did not differ significantly between LNM-negative patients based on these body composition parameters (P=0.142, P=0.118) (**Figures 5A, B**).

### 3.7 The relationship between body composition parameters and prognostic outcomes across TNM-stage subgroups

**Figure 6:**
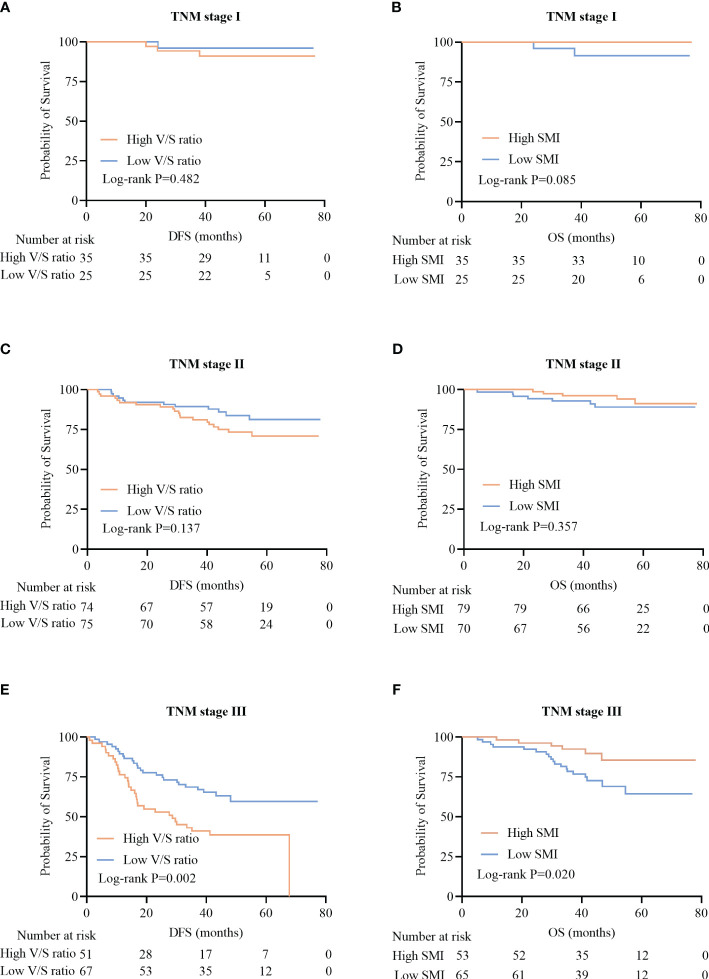
Kaplan-Meier survival curves for CRC patients with different TNM stages of disease. **(A-F)** Kaplan-Meier DFS **(A, C, E)** and OS **(B, D, F)** curves for patients with TNM stage I **(A, B)**, stage II **(C, D)**, and stage III **(E, F)** disease.

When patients were analyzed according to their TNM staging, no significant differences in the DFS of patients with TNM stage I and II disease were observed when comparing individuals with high and low V/S ratio values (P=0.482 and P=0.137) (**Figures 6A, C**). However, among TNM stage III patients, individuals with high V/S ratios exhibited a significant reduction in DFS relative to those with low V/S ratios (28.7 (13.3–41.7) vs 41.3 (25.6–49.9), P=0.002) (**Figure 6E**).

In line with these results, no significant relationship between SMI and OS was observed among patients with TNM stage I and II disease (P=0.085, P=0.357) (**Figures 6B, D**), whereas among TNM stage III patients, those individuals with a low SMI exhibited significantly worse OS than that of patients with a high SMI (31.0 (23.0–36.0) vs 32.0 (21.6–39.3), P=0.020)(**Figure 6F**).

## 4 Discussion

The present results suggest that among CRC patients with stage I-III disease, a low SMI is associated with worse OS but is unrelated to DFS, whereas a high V/S ratio is independently associated with DFS but unrelated to OS. Subgroup analyses further suggested that the prognostic benefits of SMI and V/S ratio values are more apparent in individuals with more advanced disease, including TNM stage III, T3/4, and LNM-positive patients. These results emphasize the need to assess the characteristics of skeletal muscle quality and adipose tissue distributions on diagnosis, especially in CRC patients with more advanced forms of these, thus providing an opportunity to achieve better long-term oncological outcomes.

This study included both rectal cancer and colon cancer in the analysis, primarily based on their significant similarities in biological characteristics, clinical features, and treatment strategies, with an important reason being that most rectal cancer patients in this study did not receive standard neoadjuvant therapy. This commonality provided a homogeneous basis without treatment interference, facilitating objective comparison. Moreover, the study strictly ensured the completeness of the monitoring protocol, guaranteeing accurate and reliable data. All recurrence cases (93 patients) were confirmed through outpatient records, which included regular clinical examinations and diagnostic results, accurately identifying recurrence events. For the death cases (39 patients), we employed various confirmation methods to enhance data completeness and reliability. Specifically, 30 cases were confirmed via telephone follow-up, and the remaining 9 were verified through affiliated hospital medical records. By integrating outpatient records, telephone follow-ups, and hospital records, we constructed a multi-dimensional monitoring network, reducing information omissions and misreporting.

In patients with CRC, sarcopenia has been inconsistently linked to survival outcomes, with some studies suggesting that it is significantly related to DFS and OS (13–17), whereas others have failed to ascribe any prognostic relevance to sarcopenia (18–20). These conflicting reports may partially be related to differences in ethnicity among cohorts, as sarcopenia incidences and associated threshold levels differ among ethnic groups (28). Here, SMI was found to be independently associated with CRC patient outcomes, with lower SMI values corresponding to greater mortality risk, providing support for certain past reports (29–31). Subgroup analyses specifically indicated that SMI was a more reliable predictor for CRC patients with advanced disease. This may be because patients with lower SMI values exhibit poorer chemotherapy and radiotherapy tolerance, reductions in overall treatment efficacy (32, 33), or higher complication rates while undergoing treatment (34, 35), thereby affecting their long-term prognostic outcomes. A lower SMI also tends to coincide with worse nutritional status (36), and this can substantially impact overall patient health, including their immune function and capacity for recovery, especially in individuals with advanced forms of cancer.

The degree to which fat distributions impact prognostic outcomes in CRC patients remains a matter of some debate. Higher levels of visceral fat have been linked to a greater risk of disease recurrence and death in some reports (7, 8, 21, 22), but other studies have failed to observe any relationship between these parameters, and some have even identified subcutaneous fat as a protective factor associated with a lower risk of recurrence (23, 24). Here, a high V/S ratio was found to be associated with a greater risk of recurrence, in line with what has been reported previously (37, 38). Past reports have demonstrated that subcutaneous fat exhibits a high degree of metabolic stability and greater lipolysis resistance as compared to visceral fat (39, 40), with its leptin production having a favorable impact on insulin sensitivity and overall levels of energy metabolism (41, 42). The protective effects of subcutaneous fat may thus partially overcome the adverse effects of visceral fat, thus explaining the ability of the V/S ratio, rather than visceral or subcutaneous fat tissue alone, to serve as an accurate predictor of recurrence.

The majority of adverse changes in body composition can be reversed such that there are opportunities to develop targeted interventional strategies. Prior studies have confirmed that it is possible to improve body composition, alleviate inflammation, and reduce the incidence of metabolic disorders through the use of anti-obesity medications including metformin and orlistat (43), together with lifestyle interventions such as routine physical activity and a balanced diet, thus contributing to a lower risk of cancer. In the future, studies should focus on defining new targets for therapeutic intervention so that the relationship between body composition and CRC patient prognosis can be translated into effective clinical strategies for the individualized treatment of patients.

There are some limitations to this study. For one, this was a single-center retrospective analysis that is thus susceptible to the potential for selection bias, and validation in future large-scale multi-center cohort studies will be important. In addition, only muscle area and density were evaluated in this study, without any corresponding interrogation of muscle function or strength, underscoring the need for additional assessment efforts in future prospective studies. Moreover, patients who had received neoadjuvant therapy were excluded, which may have introduced some selection bias; thus, future studies should include a more comprehensive patient population. There is also no accepted threshold for the standardized assessment of skeletal muscle or fat at present. Even so, the present results offer new evidence in support of the relevance of these parameters to oncological outcomes. While no efforts to assess intra- or inter-observer variability with respect to analyses of CT images were implemented in this study, the analytical approach employed herein has previously been shown to be objective and reliable (44, 45).

## 5 Conclusion

In summary, these data demonstrate that both the V/S ratio and SMI offer significant clinical utility as biomarkers capable of predicting long-term CRC patient outcomes. Strikingly, higher V/S ratios and lower SMI values were associated with significantly worse prognostic outcomes among CRC patients with more advanced disease. These data emphasize the need for further studies focused on the prognostic implications of different body composition parameters in CRC and their potential to guide individualized treatment efforts.

## Data availability statement

The raw data supporting the conclusions of this article will be made available by the authors, without undue reservation.

## Ethics statement

The studies involving humans were approved by Ethics Committee of Jingzhou Hospital affiliated with Yangtze University. The studies were conducted in accordance with the local legislation and institutional requirements. The ethics committee/institutional review board waived the requirement of written informed consent for participation from the participants or the participants’ legal guardians/next of kin because, as this study was retrospective in nature, the need for informed consent was waived.

## Author contributions

HZ: Conceptualization, Investigation, Methodology, Visualization, Writing – original draft, Writing – review & editing. LT: Methodology, Visualization, Writing – review & editing. YW: Investigation, Methodology, Writing – review & editing. SL: Funding acquisition, Project administration, Supervision, Writing – review & editing.
