# Supplementary material for: Computed tomography-measured body composition can predict long-term outcomes for stage I-III colorectal cancer patients
Source: Front Oncol. 2024 Jul 8;14:1420917. doi: 10.3389/fonc.2024.1420917 (PMC11260682; doi:10.3389/fonc.2024.1420917)
Supplement: Supplementary file 1 [file DataSheet_1.pdf]

frontiers

Supplementary Material

Computed tomography-measured body composition can predict long-term outcomes for stage I-III colorectal cancer patients

First Author: Han Zhou[1], Second Author: Lei Tian[1], Third Author: Yiting Wu[1]

*** Correspondence:** Corresponding Author : Sibin Liu* liusib9159@qq.com

1 Supplementary Figures and Tables

1.1 Supplementary Figures

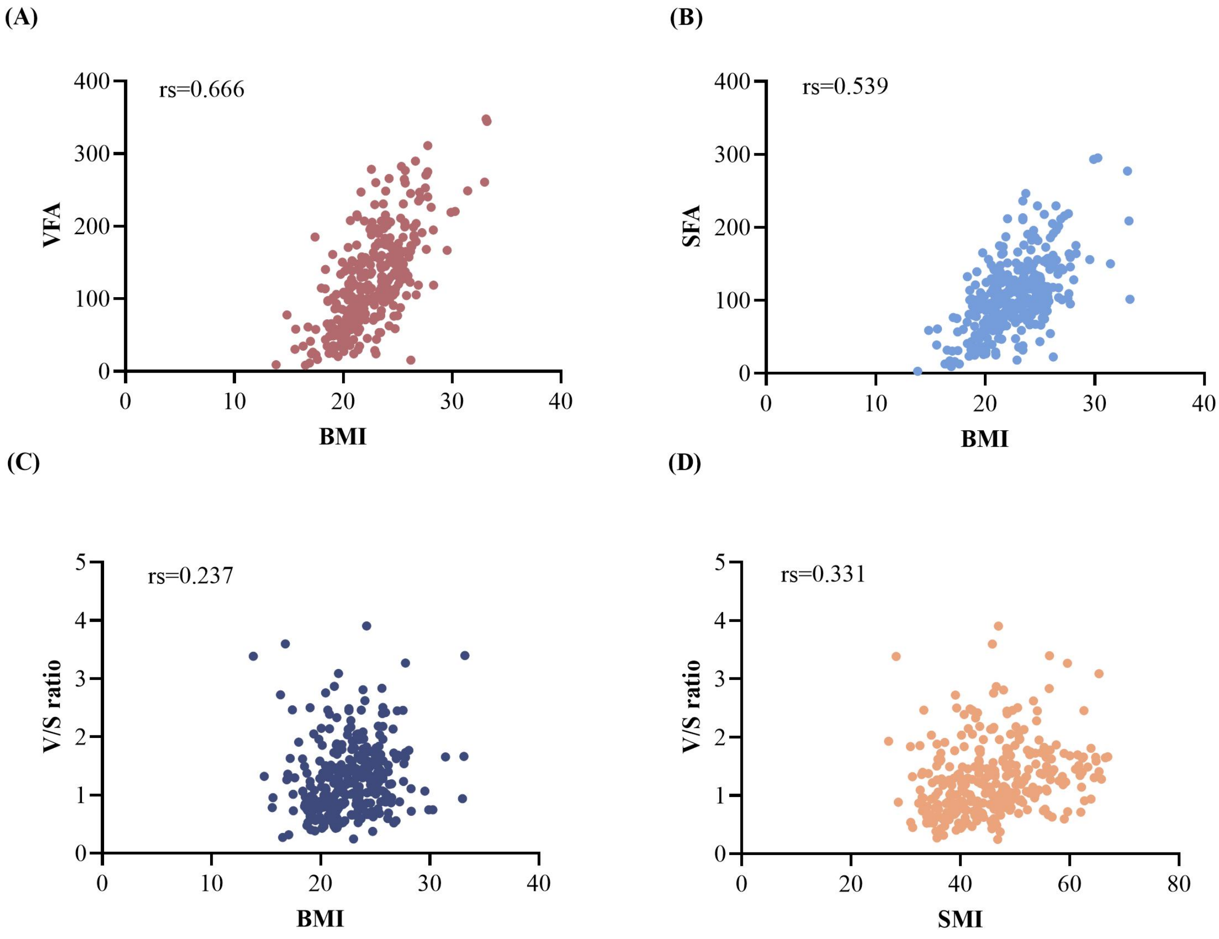

Figure S1. Correlations of body mass index(BMI) with visceral fat area(VFA) (**A**), subcutaneous fat area(SFA) (B), and the visceral-to-subcutaneous fat area(V/S) ratio (C),and the correlation of SMI with the V/S ratio (D).
